# Living with faecal incontinence: a qualitative investigation of patient experiences and preferred outcomes through semi-structured interviews

**DOI:** 10.1007/s11136-024-03756-3

**Published:** 2024-08-14

**Authors:** S. L. Assmann, D. Keszthelyi, S. O. Breukink, M. L. Kimman

**Affiliations:** 1 Department of Surgery and Colorectal Surgery, Maastricht University Medical Centre, Maastricht, The Netherlands; 2 Department of Gastroenterology-Hepatology, Maastricht University Medical Centre, Maastricht, The Netherlands; 3 Research Institute of Nutrition and Translational Research in Metabolism Metabolism (NUTRIM), Maastricht University, Maastricht, The Netherlands; 4 Research Institute for Oncology and Reproduction (GROW), Maastricht University, Maastricht, The Netherlands; 5 Department of Clinical Epidemiology and Medical Technology Assessment, Care and Public Health, Research Institute (CAPHRI), Maastricht University Medical Centre, Maastricht, The Netherlands

**Keywords:** Faecal incontinence, Quality of life, Qualitative research

## Abstract

**Purpose:**

Faecal incontinence (FI) is a prevalent and debilitating anorectal problem causing embarrassment, anxiety, and social isolation, diminishing quality of life. At present there is no optimal treatment option for FI. Consequently, treatments primarily focus on symptom reduction and improving quality of life. Understanding patient experiences and outcomes they seek from treatment is crucial for improving care. This study aims to explore how FI impacts patients’ lives and identify important treatment outcomes as part of the development of a Core Outcome Set (COS).

**Methods:**

Patients with FI were recruited from outpatient clinics in the Netherlands. Semi-structured interviews were performed, audio recorded, transcribed per verbatim and coded. Thematic analysis was performed to identify (sub)themes and categories relevant to the patients.

**Results:**

Twelve interviews were conducted before saturation was reached (75% female, 25% male, mean age 63, range 39–83 year). Four main themes emerged ‘Physical symptoms’, ‘Impact on daily life’, ‘Emotional impact’ and ‘Coping’. Patients expressed how FI severely limits daily activities and emotional wellbeing. Treatment priorities centred on resuming normal activities rather than solely on symptom reduction.

**Conclusion:**

The impact of FI extends far beyond uncontrolled loss of faeces, affecting psychological, emotional, and social wellbeing. Patients prioritise outcomes focussed on reclaiming normalcy and independence rather than focusing on physical symptoms alone. Integrating these patient-centered outcomes in future studies could enhance treatment satisfaction and patient-perceived treatment success. Furthermore, the outcomes identified in this study can be included in a Delphi survey alongside other relevant outcomes, paving the way for the development of a COS.

**Supplementary Information:**

The online version contains supplementary material available at 10.1007/s11136-024-03756-3.

## Abstract (plain language summary)

Faecal incontinence (FI), also known as unwanted bowel leakage, is a common and distressing condition that significantly impacts patients’ lives. It can cause embarrassment, worry and feelings of loneliness, significantly impairing quality of life. To better care for patients with FI, it is important to understand how they experience life with FI and what outcomes they look for in a treatment. This interview study explored patient experiences, how FI affects daily life, and identified treatment outcomes that matter most to this group of patients. The interviews revealed that patients do not just want fewer physical symptoms, but they want to regain freedom, to go out and move around like they used to, and to be able to do their daily activities without any problems.

These patient perspectives should be used in future research within this field to improve patient satisfaction and patient perceived treatment success.

**Supplementary Information:**

The online version contains supplementary material available at 10.1007/s11136-024-03756-3.

## Introduction

Faecal incontinence (FI), as per the Rome IV criteria, is defined as the ‘‘recurrent uncontrolled passage of faecal material for at least 3 months’’ [1]. FI is a global issue impacting people across all income levels and countries and it is estimated that approximately 7.7% of the global population suffers from this debilitating anorectal problem [2, 3]. Continence refers to the ability to control bodily fluids, such as urination and defecation. Incontinence, on the other hand, is the lack of control over these functions. There are different types of incontinence. Urine incontinence involves the involuntary leakage of urine, while double incontinence refers to the simultaneous occurrence of both urine and FI. FI, the focus of this study can be caused when any of the following factors or the interaction between them fails: the anal sphincter complex, rectal reservoir function, neurological function and stool consistency [4].

While a singular event like a major anorectal injury can cause FI, it often results from a combination of factors such as advancing age, obstetric injury, menopause, rectal inflammation, diseases of the nervous system, obesity, chronic illness and unknown contributors [1, 5, 6].

Patients with FI are frequently embarrassed and anxious about the possibility of losing faeces in public, leading to visible leaks or unpleasant odors. Additionally, many are reluctant to disclose their condition to other. This can result in low self-esteem, avoidance of activities, social isolation and depression. Consequently, this can have a detrimental impact on the quality of life (QoL) of the patient [7–10].

Only in a small number of patients, FI treatment(s) will result in the complete elimination of uncontrolled faecal material [11]. The management of patient with FI therefore not only prioritizes the pursuit of a cure, but also emphasises supporting the patient and their lifestyle.

The success of a treatment option is largely contingent on the patient’s subjective experience of life with FI, their primary complaints and the impact on their QoL. This extends beyond physical and mental QoL to encompass an individual’s perception of their position in life, considering cultural and value systems in which they live [12]. It also includes, goals, expectations, concerns and the specific outcome the individual patient aims to achieve from their treatment.

There is a scarcity of comprehensive assessments detailing the lived experiences of patients who suffer from FI. Qualitative studies related to patient experiences with FI reveal that the burden of disease for the condition extends far, significantly impacting the overall well-being of affected patients [13–16]. Furthermore, there is a lack of studies investigating the treatment preferences of patients with FI or the specific treatment outcomes they desire for managing this condition. Such knowledge would not only benefit healthcare professionals in providing more effective support for these patients but also enable the identification of outcomes crucial to patients’ well-being. The insights derived from patient interviews can serve as valuable input for developing a Core Outcome Set (COS) [17]. A COS is an agreed minimal set of outcomes which must be measured and reported in all clinical trials within a specific domain of health or healthcare [17]. The COMET initiative outlines several steps for the creation of a COS, after the scope has been determined, the project has been registered and a protocol has been created, ‘what to measure’ should be determined [17]. This involves identifying existing knowledge regarding potential outcomes while also addressing any gaps within it. Drawing on the experiences of patients with FI can help fill these gaps in research [17, 18]. Incorporating patient-centred outcomes into a COS will guarantee that future clinical studies prioritise the needs and preferences of patients. Hence, the objective of this study is twofold: to gain a deeper insight into the lived experiences of patients suffering from FI in The Netherlands, a high-income country and secondly, to identify the treatment outcomes they prioritise as part of the development of a COS.

## Methods

### Study design and participants

#### Qualitative approach

We employed a qualitative approach using semi-structured interviews designed to explore detailed descriptions of patient experiences related to FI. Participants were recruited from the Surgery and Gastroenterology outpatient clinics of Maastricht University Medical Center+ (MUMC+) and through an MUMC + database of patients who have previously granted permission to be contacted. Eligible participants met Rome IV criteria for FI, were aged 18–85 years, and were purposively sampled for gender, age, and treatment stage to ensure representation and diversity within the FI population. The sample size was determined by the point at which saturation was reached, defined as the point at which no new categories arose over three consecutive interviews [19].

The COREQ (COnsolidated criteria for Reporting Qualitative research) checklist was used (See Online Resource 1).

### Data collection methods

An interview guide, developed and pilot-tested with input from clinicians and methodologists, included 10 core questions addressing FI onset, impacts on daily life, coping mechanisms, received treatments, and treatment outcomes (see Online Resource 2). Interviews were conducted either in-person at MUMC + or via video calls, and were led by a researcher experienced in FI research but with no prior relationship with the participants. All interviews were audio recorded, transcribed verbatim, and coded using an inductive approach to identify themes and patterns in the data.

### Data analysis

Using the framework method with an inductive open coding approach, transcripts were independently analyzed by two researchers (S.A. and M.K.) in the Atlas.ti application to uncover themes and categories emergent from the data. Initial coding discrepancies were resolved through team discussion, ensuring analytical rigor and consistency across interpretations [20]. The final coding framework was reapplied to previously coded transcripts.

For detailed information on additional methodological specifics and the study authors, please refer to Online Resource 3 and 4 respectively.

## Results

Twelve patients (9 females, 3 males, aged 39 to 83 years) received the patient information sheet for this study, and all agreed to participate in the interviews. The interviews were conducted between November 2022 and March 2024.

While the majority of participants were unable to pinpoint an exact date of onset for their incontinence symptoms, given their varied manifestation and progression, patients reported experiencing FI for durations ranging from half a year to approximately 50 years (mean duration of 17 years) each at different stages in their treatment pathway.

FI in these patients stemmed from anorectal trauma due to surgery, obstetric anorectal trauma and multifactorial causes. All patients had tried multiple treatment options as shown in Table 1. Interview duration ranged between 22 and 58 min. During the tenth, eleventh and twelfth interview, no additional categories emerged and thus our stop criterion for data saturation had been reached.

**Fig. 1:**
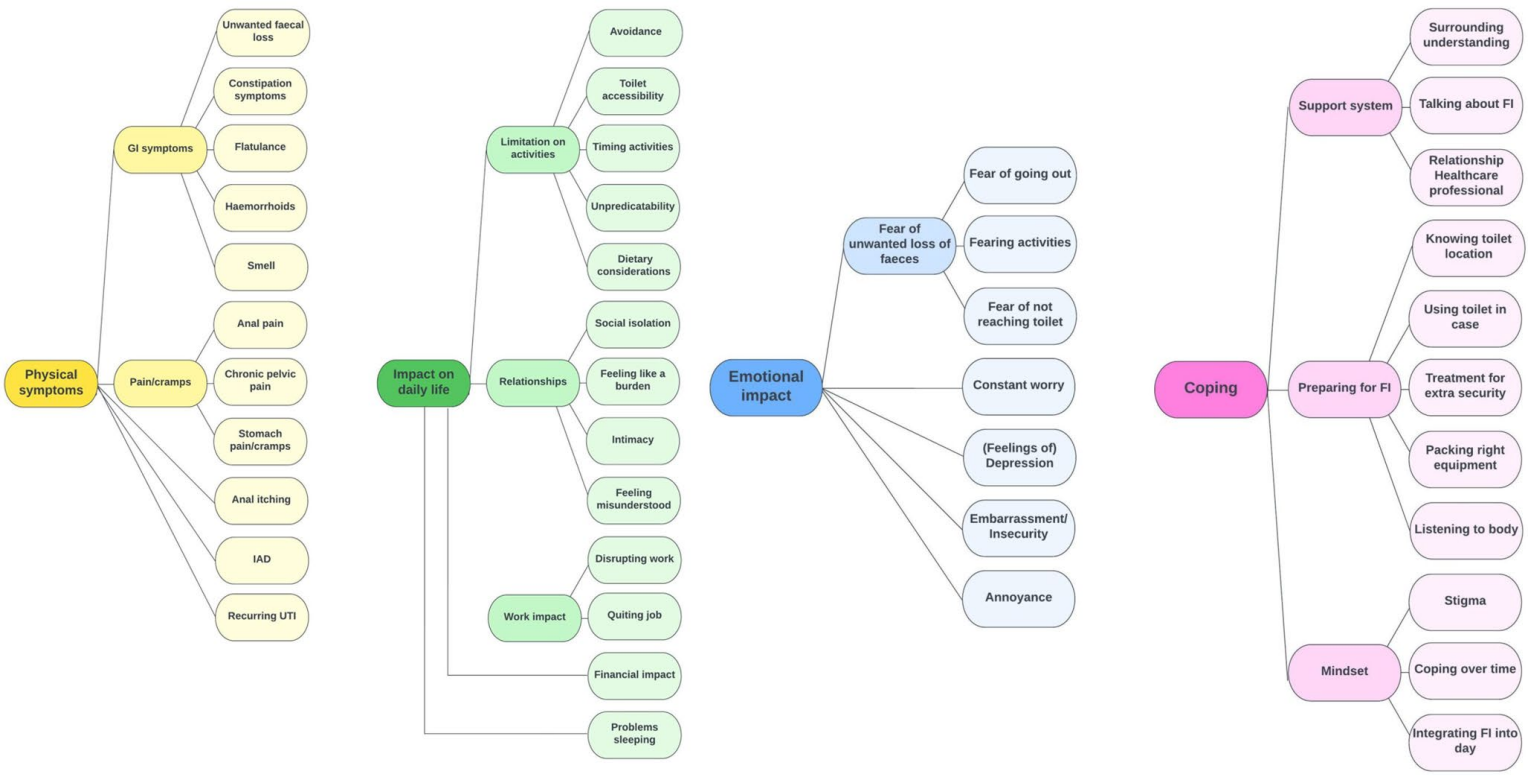
Central (sub-) themes and categories identified GI = gastrointestinal, IAD = incontinence associated dermatitis, UTI = urinary tract infection, FI = faecal incontinence

Four main themes were identified through patient interviews: ‘Physical symptoms’, ‘Impact on daily life’, ‘Emotional impact’ and ‘Coping’. The (sub)themes and categories identified within this framework are outlined in Fig. 1, accompanied by illustrative quotes detailed in Table 2. Additionally, patients provided key patient centric factors related to FI treatment, encompassing positive treatment effects, important aspects surrounding a treatment and desired treatment outcomes, shown in Table 3.

**Table 1:** Patient characteristics

Patient characteristics	*n* = 12
Age (years), mean (range)	66 (39–83)
Female, n (%) Male, n (%)	9 (75%) 3 (25%)
Causes, n (%)	
Multifactorial	8 (67%)
Anorectal trauma after surgery	4 (33%)
Treatment, n (%)	
Incontinence materials*	10 (83%)
Loperamide	7 (58%)
Psyllium husk	4 (33%)
Pelvic floor physiotherapy	8 (66%)
Rectal irrigation	6 (50%)
Sacral neuromodulation	4 (33%)
Laxatives	1 (8%)
Anal plug	1 (8%)

*Incontinence material includes diapers, menstrual/incontinence pads, toilet paper, incontinence pants

**Table 2:** Themes and categories with illustrative quotes

Themes	Category	Quote
Physical symptoms	GI symptoms	*‘’I wasn’t able to hold it in and it* [faeces] *just ran down my legs in the middle of a busy city’’.* *-*Female, 66
		*‘’It’s just that I can’t make it to the toilet in time*,* or I’m too late’’-* Female, 39
	Pain/cramps	*‘’The anal pain did wake me up*,* it would take fifteen minutes before the cramps or the pain were gone’’* -Female, 59
	Other	*‘’I suffer a lot from flatulence’’* -Female, 66
Impact on daily life	Limitation on activities	‘*’Suppose I have to be somewhere at 9 o’clock*,* then I already know I have to get up at 5 so that I can use the toilet properly*’’ -Female, 65
		*‘’I need to think about how it feels on that day and then decide whether we can go* [out] *or not’’* -Female, 59
		‘*’And many activities I have simply given up on’’* -Female, 64
	Relationships	*‘’ But you also have the sexual aspect… And then you think*,* yes*,* okay*,* but first* [I’ll] *check in the bathroom to see if anything* [FI episode] *has happened.’’* Female, 65
	Work impact	‘’ *I had to give up on my volunteer work because I worked with small children*,* because yea*,* you can’t just leave them on their own*’’ [when needing the toilet or cleaning yourself up] -Female, 64
	Other	‘*’My husband would wake me up* [after an FI episode], *and then I would switch on the shower and I would try to go back to sleep*,* but I wouldn’t be able to get back to sleep’*’-Female, 69
Emotional impact	Fear of unwanted loss of faeces	‘*’When I’m not at home I get this fearful*,* anxious feeling*’’ -Female, 74
	Other	’*It impacts your whole life*,* you have to constantly take into account that that* [FI episode] *can happen*’’ -Female, 83
Coping	Support system	‘*’The girls are like ‘Oh mom*,* we’ll have a coffee*,* you can use the toilet and then we’ll figure out the next part’ So in that regard they take it into consideration’’* -Female, 59
		*‘’And sometimes I’m late* [for work] *they understand that’’-* Female, 65
	Preparing for FI	‘*’When I go to the city*,* I search for the nearest toilet… Then I go from one toilet to the next*.’’ -Male, 60
		*‘’Making sure I have a lot of incontinence materials in my bag so that I’m prepared for anything.’’* -Female, 64
	Mindset	*‘’At some point you have to overcome that hurdle: I have this [FI]*,* I have to learn to live with it’’* -Female, 69

### Physical symptoms

The theme ‘Physical symptoms’ encompassed three categories. Among these ‘Gastrointestinal symptoms’ covered various aspects. When addressing the primary symptom of FI, specifically the involuntary loss of faeces, patients emphasized several key points. These included the frequent urgency to use the toilet, the unpredictable nature of FI, and the inability to control bowel movements. Patients also reported a lack of sensation to defecate and absence of awareness of faecal leakage. The urgency to reach the toilet promptly and subsequently not reaching the toilet on time were recurrent topics of discussion: *‘’I wasn’t able to hold it in and it* [faeces] *just ran down my legs in the middle of a busy city’’* (female, 66). Some patients highlighted that the severity of their FI increased during exercise and illness with some indicating an increased severity when feeling nervous or experiencing urinary tract infections. Additionally, many patients reported experiencing other gastrointestinal symptoms such as constipation, increased flatulence, smell due to faeces, and haemorrhoids.

Furthermore, within the category ‘Pain and cramps’, patients described a range of discomfort, including sudden anal pain, chronic pelvic pain, and stomach pains and cramps.

Other physical symptoms identified were broken skin around the anus, often attributed to prolonged contact with faeces (i.e. incontinence associated dermatitis). This was associated by a lack of awareness regarding faecal leakage or the inability to promptly clean themselves. Additionally, patients described anal itching, particularly exacerbated on days with increased faecal loss and recurring urinary tract infections.

### Impact on daily life

The theme ‘Impact on daily life’ included three categories. Within the ‘Limitation on activities’ category, patients discussed experiencing a significant disruption in their ability to lead their lives as they desire, often avoiding various activities as a result: ‘*’And many activities I have simply given up on’’* (Female, 64). One specific activity patients mentioned they avoided is exercise, particularly due to its exacerbating effect on symptoms. Additionally, patients expressed their inability to engage in hobbies when access to toilets was limited, or attend social gathering where frequent bathroom visits would be noticeable. The latter scenario often resulted in feelings of embarrassment.

Patients disclosed a tendency to stay at home when possible. Many noted a decline in spontaneous outings, attributing it to the need to prepare their bowels before leaving home, or limiting outings to periods of minimal symptoms. Patients expressed frustration over the unpredictability of FI, finding it challenging to plan ahead, without knowing the severity of their symptoms on a given day. Additionally, patients discussed adjusting their diets to avoid an exacerbation in symptoms, and some refrained from dining out entirely.

Furthermore, the category ‘relationships’ was identified, highlighting a significant impact on patients’ social interactions following the onset of FI. Many patients reported a noticeable decline, reflected in their reluctance to visit their friends’ homes and engaging in fewer activities outside of the home with friends and family. Some expressed feeling anxious around people due to their FI, noting that these feelings could potentially further exacerbate symptoms. Patients described feeling like a burden to others around them, recognising that friends and family often need to adjust their plans to provide support, while also recognising their own role in frequently occupying the toilet and the potential discomfort others may experience due to the odour of the faeces. Additionally, patients highlighted the negative effects FI can impose on intimacy with their partners, including its impact on their sexual life. They also noted that their friends and family frequently overlook their FI, as it is not a visible limitation, or fail to grasp the severity of the issue, resulting in feelings of being misunderstood. However, it’s noteworthy that many patients felt well-supported by their friends and family, as elaborated upon under the subheading ‘coping’.

Other life impacts discussed by patients included requiring time away from work due to frequent bathroom trips or the need to clean themselves. Some patients expressed contemplating resignation from employment due to the severity of their condition. Furthermore, patients discussed the financial strain stemming from treatments and medical appointments. Sleeping difficulties arising from faecal loss, pain, or cramps were also prevalent concerns among patients.

### Emotional impact

The theme ‘Emotional impact’ consisted of two categories. ‘Fear of unwanted loss of faeces’.

emerged as the prevailing emotion during the interviews. The concern over unwanted loss of faeces was highlighted as a significant factor affecting the lives of many patients.

Fear of leaving home, engaging in certain activities and not reaching the toilet in time were often discussed. The ‘Other emotional impact’ all patients expressed was a constant worry caused by FI, emphasising its persistent disruption into their daily lives: ’*It impacts your whole life*,* you have to constantly take into account that that* [FI episode] *can happen*’’ (Female, 83). Feelings of embarrassment and insecurity stemming from loss of faeces were commonly mentioned. Moreover, the broader emotional toll of FI was discussed, with some acknowledging experiencing depressive feelings and heightened feelings of annoyance due to their symptoms.

### Coping

The theme ‘Coping’ encompassed three themes. Within the theme ‘Support system’.

the support patients receive from understanding friends and family members who assist them in various ways was highlighted. This support includes reminders to take medications before leaving, organizing activities with accessible toilet facilities, and respecting their need for privacy. Engaging in conversations about FI with loved ones, as well as educating others facing similar challenges, proved beneficial for some individuals in coping with the condition.

The relationship with healthcare professionals treating FI emerged as a significant factor for several patients. While some expressed dissatisfaction due to feeling unheard, lacking compassion, or receiving insufficient explanations about FI initially, others reported positive experiences characterized by feeling understood and taken seriously.

Additionally, patients also voiced a need for earlier discussions with their healthcare professional regarding treatment options, including incontinence products, to help manage the condition. They noted that these discussions often occurred much later than desired, sometimes not taking place at all.

Another theme often mentioned was ‘Preparing for FI’. All patients share a common practice of consistently seeking out nearby toilets in anticipation of potential defecation needs, often pre-emptively using the toilet before departing. Additionally, some patients mentioned resorting to rectal irrigation and utilizing incontinence materials (i.e. diapers, menstrual/incontinence pads, toilet paper, incontinence pants) as precautionary measures.

Moreover, patients discussed the importance of carrying supplies in case of unwanted loss of faeces, including incontinence materials, medication, spare clothing, wet wipes and other cleaning items. Another coping strategy for some patients involved listening to their body cues and timing outings during periods when FI severity was low.

Furthermore, the theme ‘Mindset’ was discussed. Patients commonly perceive a significant stigma surrounding FI, with several noting that fellow sufferers often hesitate to discuss their issues and may even avoid seeking medical assistance due to shame or fear. However, many patients reported an improvement in coping with FI over time, with some attributing this to a change in mindset: *‘’At some point you have to overcome that hurdle: I have this [FI]*,* I have to learn to live with it’’* (Female, 69). Over time, feelings of embarrassment and avoidance of activities appeared to diminish in some. Patients also found strategies to manage FI and incorporate it into their daily routines. For instance, some engaged in normal daily activities such as reading or solving crosswords while simultaneously using time-consuming treatment options (i.e. rectal irrigation, for which patients often need to sit on the toilet for 30–60 min).

### Patient insights on treatment priorities

Throughout the interviews, patients highlighted several positive effects they had experienced from treatment options they had tried, including the ability to delay defecation, the ability to resume daily activities, feeling more confident and an observed improvement in social interactions.

Patients also highlighted which aspects of a treatment and its surrounding factors are important to them as shown in Table 3. These include a good relationship with the healthcare professional, feeling respected and heard and receiving personalised care, as well as maintaining a sense of autonomy ‘’The fact that I can manage it myself… I’m kind of a director of my own disease progress… It is very pleasant that I can have a bit of control over it myself’’ (female, 39). Additionally, patients expressed preferences for treatment options that are comfortable, minimally disruptive to daily life, and not overly time consuming. Some also noted the importance of treatment options tailored specifically to FI, referring to incontinence products which are currently often designed to address urinary rather than FI.

Patients deliberated on treatment outcomes they seek, encompassing reductions in symptoms such as loss of faeces, needing to use the toilet, diarrhoea and stomach pain, as well as the freedom to do activities, and to resume various activities such as exercising, and engaging in social interactions. They also expressed a desire for the ability to partake in spontaneous endeavours, where they could readily participate without the need for preparation. Additionally, improvement in sleep quality was highlighted as another important outcome sought by patients. Furthermore, patients expressed desires for emotional well-being, including, reduced fear, an increased sense of security, more fun in life and a better peace of mind. Some patients also emphasised an enhancement in their sexual life as a desired outcome, noting that it had been negatively affected by the consequences of FI.

**Table 3:** Patient important treatment factors

Important treatment factors	Illustrative Quote
Relationship HCP	***‘’****What’s also important to me is having trust in my healthcare provider…It’s important to me that they understand what I’m trying to say.’’* (female, 64)
Individualised approach	*‘’I really liked… that they considered what* [treatment] *would best taking into consideration my family life and my work situation’’* (female, 39)
Patient in control	*‘’ I want to decide for myself when to take one* [Loperamide]. *If I take 5* [as recommended] *I won’t feel like I’m in control anymore.’’* (female, 74)
Minimal impact on life	*‘’Because I have three young children and work irregular shift*,* it’s nice not to have to worry about that* [impact on daily life] *too*,* so I find it very important’’* (female, 39)
Time consumption	*‘’Because if you have to get up at 5:30 to be able to get to work at 7:30 to empty your bowels*,* it’s not really feasible for someone who has a job.’’* (female, 66)
Treatment FI specific*	*‘’Pads aren’t made for faeces. There are these pads*,* they have the extra long ones*,* but then I have to use two in a row because one is too small. I mean*,* if you have an accident*,* the won’t catch it*,* obviously’’* (female, 65)
Treatment not too uncomfortable	*‘’I found it too uncomfortable. I couldn’t get it [rectal irrigation system] in and*,* the nozzle was also a bit hard.’’* (male, 82)

HCP = Healthcare professional

*Treatment specific for FI: e.g. incontinence products which are specifically designed for faecal incontinence rather than urinary incontinence or menstruation

## Discussion

This pragmatic qualitative study comprised twelve semi-structured interviews conducted with patients experiencing FI. These interviews provided a deeper insight into the lived experiences of these patients, unveiling four primary themes: ‘Physical symptoms’, ‘Impact on daily life’, ‘Emotional impact’ and ‘Coping’. Additionally, the interviews identified 15 desired treatment outcomes proposed by patients.

This study has demonstrated that FI significantly affects patients in ways that extend far beyond the physical symptom of unwanted stool loss alone, as has been shown in a number of other qualitative studies regarding FI [13–16]. During the interviews in this study, many patients noted experiencing additional physical symptoms, including gastrointestinal problems, pains, cramps, incontinence associated dermatitis, recurring urinary tract infections and anal itching. However, these physical symptoms constitute only a fraction of the challenge’s patients endure. Beyond physical discomfort, FI profoundly disrupts patients’ day-to-day lives. This encompasses limitations on activities, strained relationships with friends and family, work disruptions and financial burdens. Moreover, these challenges impose a significant emotional toll, inducing fear of accidents, persistent worry, feelings of embarrassment, depression, annoyance and negatively impacting their sexual well-being.

The strategy patients employ to manage and cope with FI also significantly influences their daily lives. Constantly needing to anticipate unwanted stool loss prompts behaviours such as familiarising themselves with toilet locations, ensuring they have used the toilet before leaving, packing necessary equipment, utilising (time-consuming) treatment options for added security. The majority of the (sub)themes have been discussed in literature previously. Notably, always knowing the nearest toilet and staying close to one, wherever patients go, is a commonly emphasized strategy in FI studies [13–16].

Beyond the above-named challenges associated with FI, patients may also struggle with the stigma surrounding this condition. Some noted that fellow FI patients are often reluctant to discuss their issues openly and may even refrain from seeking medical help due to feelings of shame or fear, which is in line with previously documented studies [13, 15]. However, notably, the interviewed patients expressed finding it beneficial to share their experiences of living with FI with others, with the majority no longer feeling embarrassed to talk about their condition. This finding appears contradictory when compared to other reported interviews but may be explained by cultural differences, as Dutch people tend be quite direct compared to Swedish and English patients [15, 16].

Aligned with these studies, our findings indicate that patients prioritise restoring normalcy across all aspects of their lives, rather than solely aiming for a reduction in FI episodes [13–16]. Participants in our study emphasised the emotional aspect of FI, particularly the feelings of fear and insecurity, to a greater extent than documented in previous studies [13–16]. Although this study confirms many themes which have been described in the literature previously, this study has also resulted in new insights into outcomes patients seek in a treatment for FI. A significant portion of the outcomes mentioned centred around the non-physical aspects of the condition, such as the ability to engage in various activities such as exercising, socialising, pursuing spontaneous endeavours and maintaining intimacy. Additionally, patients expressed a strong desire for emotional well-being, including, reduced fear, and an increased sense of security. While studies in the FI field often incorporate outcomes related to non-physical symptoms, such QoL, the emphasis placed on physical outcome like ‘episodes of FI’ and ‘severity of FI’ in clinical research typically outweighs the consideration given to non-physical outcomes such as QoL and emotional well-being [11]. However, our study reveals a contrasting perspective, with patients emphasizing the importance of non-physical outcomes and not only physical ones. Established QoL assessments, such as the Faecal Incontinence Quality of Life Scale (FIQL), cover several aspects emphasised as important during these patient interviews [21].

In clinical trials, where QoL serves as an outcome and the FIQL or a similar QoL instrument is employed, the patient-important items, such as avoiding certain activities and feelings of fear, comprise only a small component of a single outcome. If indeed patients deem many of these items as crucial when evaluating the effectiveness of a treatment option, the way forward may be for physicians and researchers to address these outcomes individually rather than as part of an average QoL score.

The insights shared by participants in this study combined with outcomes identified in the literature, can serve as a valuable input for the development of a COS [22]. To determine the prioritisation of outcomes by patients, physicians and clinical researchers, the outcomes identified in this study and through literature review will be put forward in a Delphi survey. Subsequently, this survey, followed by a consensus meeting will lead to a COS for the evaluation of FI treatment [22].

This study has some limitations. Although this study aims to enhance credibility and therefore trustworthiness by defining clear inclusion and exclusion criteria, selecting a representative sample of the FI population, using pilot-tested semi-structured interviews, and being transparent about the background characteristics of the research team, trustworthiness could have been further improved by incorporating member checking, allowing participants to verify the findings [23]. Additionally, incorporating other data sources such as input from partners, family or close friends and triangulating the data may have increased trustworthiness [23].

It is also important to acknowledge that the patients who voluntarily engage in interviews to discuss their experiences with FI are those comfortable discussing their condition. Consequently, the inclusion of patients who are less inclined to discuss their condition may yield different thematic insights. Additionally, participants were recruited from a hospital setting, which may bias the sample towards individuals proactive in seeking help for their FI and likely to have explored several treatment options. It is plausible that different themes may have emerged among patients who have not yet sought any help or treatment for their condition. Furthermore, recruitment of females proved much easier than recruitment of males. However, this recruitment approach from a hospital setting and the gender discrepancy were anticipated and reflect the demographics typically seen in clinical trials. Therefore, this is a suitable population for identifying potential outcomes which could be included in a COS. Lastly, only participants from the Netherlands were included in this study, potentially neglecting insight into how cultural differences in other societies impact the experience of living with FI, specifically low- and middle-income countries.

## Conclusion

This qualitative study offers profound insights into the lived experiences of individuals coping with FI and showed which outcomes patients prioritise to manage their condition. The findings show that the impact of FI extends far beyond uncontrolled loss of faeces, affecting psychological, emotional, and social wellbeing. Patients prioritise outcomes focussed on reclaiming normalcy and independence, emphasising a holistic approach beyond addressing physical symptoms alone. Integrating these patient-centered outcomes into future studies has the potential to enhance treatment satisfaction and patient-perceived treatment success.

To achieve this, the next step involves presenting the outcomes identified through interviews in this study, along with those identified from a literature review, in a Delphi survey involving relevant stakeholders [24]. Subsequently, a consensus meeting will take place to determine a finalised list of outcomes to be incorporated into a COS for FI.

## Electronic supplementary material

Below is the link to the electronic supplementary material.

Supplementary Material 1

Supplementary Material 2

Supplementary Material 3

Supplementary Material 4
