# Supplementary material for: Living with faecal incontinence: a qualitative investigation of patient experiences and preferred outcomes through semi-structured interviews
Source: Qual Life Res. 2024 Aug 14;33(11):3121–9. doi: 10.1007/s11136-024-03756-3 (PMC11541390; doi:10.1007/s11136-024-03756-3)
Supplement: Supplementary file 2 — Supplementary Material 2 [file 11136_2024_3756_MOESM2_ESM.docx]

**Article title:** Living with Faecal Incontinence: A qualitative investigation of patients experiences and preferred outcomes through semi-structured interviews

**Journal name:** Quality of Life research

**Author names:** S.L. Assmann, S.O. Breukink , D. Keszthelyi, M.L. Kimman

**Corresponding author:** S.L. Assmann, [s.assmann@maastrichtuniversity.nl](mailto:s.assmann@maastrichtuniversity.nl), Maastricht University, The Netherlands

**Online resource 2: Interview guide (Translated from Dutch)**

Prior to interview

- Go over the purpose of the study
- Determine willingness to take part in the study
- Check if participant agrees for the interview to be audio-recorded, stored and used for presenting research findings anonymously.
- Ask if there are any questions regarding the interview procedure
- Ask participant to complete the informed consent form if not already completed.

Part 1: Patient experiences of living with Faecal incontinence

**1. When were you first diagnosed with involuntary loss of faeces? Can you tell us more about this?**

**2. What complaints related to involuntary loss of faeces do you experience?** (e.g. Stool loss, abdominal pain, diarrhea etc.)

**3. How does involuntary loss of faeces impact your daily life** (e.g. limitations)?

- Do you avoid certain situations (e.g. meeting friends, travelling, shopping, eating out)?

- Are you concerned about not reaching the toilet on time?

- Do you feel any sense of shame due to your involuntary stool loss?

**4. How do you manage you involuntary loss of faeces complaints?**

- Do you try to stay close to a toilet?

- Do you adjust your schedule according to your stool patterns?

- How does your social circle react to your stool loss, if they are aware of it?

- Does your stool loss affect the lives of those close to you?

- Do you notice any social stigmas associated with involuntary loss of faeces? How does that make you feel?

Part 2: Treatment

**5. What therapies/treatments have you received so far?**

- What are your experiences with these various treatments?

**6.** **What is the most important aspect of a treatment for you?** What impact do you want the treatment to have on your stool loss, on the complaints you experience and/or on your daily life?

**7. Why did you choose the current treatment you are receiving?** How much input did you have in selecting this treatment?

- Have you ever had to pay for a treatment?

**8. What do you know about the different treatment options available for involuntary loss of faeces?**

**9. What characteristics do you find important in FI treatment options, and why?** (e.g. travel convenience, costs)

Part 3. Closing

**10. Is there anything else you would like to share?** What should healthcare professionals know to better assist patients with involuntary loss of faeces?

Lastly, do you have any questions for us?
