# Supplementary material for: Living with faecal incontinence: a qualitative investigation of patient experiences and preferred outcomes through semi-structured interviews
Source: Qual Life Res. 2024 Aug 14;33(11):3121–9. doi: 10.1007/s11136-024-03756-3 (PMC11541390; doi:10.1007/s11136-024-03756-3)
Supplement: Supplementary file 3 — Supplementary Material 3 [file 11136_2024_3756_MOESM3_ESM.docx]

**Article title:** Living with Faecal Incontinence: A qualitative investigation of patients experiences and preferred outcomes through semi-structured interviews

**Journal name:** Quality of Life research

**Author names:** S.L. Assmann, S.O. Breukink , D. Keszthelyi, M.L. Kimman

**Corresponding author:** S.L. Assmann, [s.assmann@maastrichtuniversity.nl](mailto:s.assmann@maastrichtuniversity.nl), Maastricht University, The Netherlands

# **Online Resource 3: Detailed method of study**

# **Methods**

**Study Design and participants**

## Qualitative approach

Qualitative semi-structured interviews were conducted using a pragmatic approach, which prioritizes practicality and seeks to comprehend phenomena, processes, or participants perspectives by exploring detailed descriptions provided by participants[1]. The interviews were semi-structured to allow the interviewer to guide the topics covered, whilst still ensuring any subjects important to the patient were discussed.

Participants were recruited through the Surgery and Gastroenterology outpatient clinics of the Maastricht University Medical Center+ (MUMC+). Additionally, patients with FI who had previously granted permission to be contacted for research purposes were recruited from a MUMC+ database.

Recruitment in the outpatient clinic was facilitated by the attending healthcare professional, who enquired whether the patients were interested in participating in this study. Patients expressing an interest were directed to the adjacent room, where the researcher provided more detailed information about the study, the patient information sheet, consent form and contact information for further inquiries. Recruitment via the patient database involved emailing the patients an information sheet accompanied by the question whether they would be interested in participating in the study.

Patients with FI according to the Rome IV criteria (recurrent uncontrolled passage of faecal material for at least 3 months) and aged between 18 and 85 year were eligible to participate[2]. Exclusion criteria were insufficient command of the Dutch language, insufficient cognitive function to answer interview questions, prior diagnosis of inflammatory bowel disease (IBD), patients with FI primarily caused by neurological disease and nursing home patients. Purposive sampling was used, taking into account gender, age and point in treatment pathway, in order to ensure the FI population was well represented, capturing heterogeneity, whilst including patients critical for the study [1].

The sample size was determined by the point at which saturation was reached, defined as the point at which no new categories arose over three consecutive interviews, a stopping criterion often used in qualitative research[3].

The COREQ (COnsolidated criteria for Reporting Qualitative research) checklist was used (See Online Resource 1).

## Data collection methods

An interview guide was constructed by the research team (clinicians and methodologist) and pilot tested among four patients and optimised prior to the start of the first interview. The topic guide was created guided by literature review and clinical expert opinion and included 10 questions regarding the initial onset of FI, its impact on the participant’s life, how (s)he copes with FI and which treatment(s) were received, and what s(he) perceives as important outcomes of a treatment (i.e. when a treatment is successful or effective). Additionally, the topic guide included possible follow-up questions designed to encourage participants to share more if they did not do so initially. The topic guide was created to guide the conversation in a constructive direction, facilitating participants in articulating their own experiences and discussing matters they considered important(see Online Resource 2).

The interviews were conducted either in a dedicated outpatient clinic room at MUMC+ or through video calls using the Teams application, based on the individual preferences of the participant. Each participant was interviewed once. Interviews were conducted by a researcher with experience in the FI field (SA). To minimize bias, the interviewer had no prior clinical relationship with any of the participants. The interviewer was trained by a qualitative researcher (MK) who also oversaw creation of the topic guide and data analysis (see appendix 2 for details about the authors).

All interviews were audio recorded onto a device specifically used for research purposes. Participants signed the informed consent form prior to the recording, at which time they were also reminded to avoid talking about any identifiable information. Each interview was transcribed verbatim and assigned a numerical code for participant identification. The key for this code was kept in a password protected excel file. Each interview was transcribed aided by Microsoft Word shortly after each interview took place. Consequently, field notes were considered redundant. Interviews were deleted from the recording device once they were transcribed.

## Data analysis

Given the exploratory nature of this study, focused on uncovering new insights, we used the framework method with an inductive open coding approach, as described by Ritchie et al. This method facilitated a thematic analysis of the interviews, outside of a pre-existing theoretical framework, enabling the organic emergence of themes and patterns from the data[4].

To enhance familiarity with the content of the interviews, each transcript was thoroughly read in full before the coding process commenced. The first three interviews were coded independently by two different researchers using the Atlas.ti application. Given that personal and professional experiences, along with pre-study beliefs, can influence the analysis process, the research team included members with various backgrounds and perspectives to address this challenge. A detailed description of the research team’s background can be found in appendix 2. The coding from the two researchers was compared and used to identify preliminary categories. Subsequently, these categories were grouped into themes and possibly subthemes in Microsoft Excel, creating an adaptable analytical framework. This framework remained dynamic throughout the analysis of each interview, allowing for adjustments as needed. Subsequent transcripts were analysed and coded by one researcher using this preliminary framework. Any new codes and categories identified were added to the framework. To ensure the framework's consistency, once the framework was finalized, it was reapplied to all previously coded transcripts to ensure uniformity and comprehensiveness. This step ensured that all data were indexed using the final framework.

**References**

1. Savin-Baden, M., & Howell Major, C. (2013). Qualitative research The essential guide to theory and practice. USA and Canada: Routledge.

2. Rao, S. S., Bharucha, A. E., Chiarioni, G., Felt-Bersma, R., Knowles, C., Malcolm, A., & Wald, A. (2016). Anorectal disorders. Gastroenterology, 150(6), 1430-1442. e1434.

3. Hennink, M., & Kaiser, B. N. (2022). Sample sizes for saturation in qualitative research: A systematic review of empirical tests. Social science & medicine, 292, 114523.

4. Ritchie, J., Lewis, J., Nicholls, C. M., & Ormston, R. (2013). Qualitative research practice: A guide for social science students and researchers: sage.
