# Supplementary material for: Living with faecal incontinence: a qualitative investigation of patient experiences and preferred outcomes through semi-structured interviews
Source: Qual Life Res. 2024 Aug 14;33(11):3121–9. doi: 10.1007/s11136-024-03756-3 (PMC11541390; doi:10.1007/s11136-024-03756-3)
Supplement: Supplementary file 4 — Supplementary Material 4 [file 11136_2024_3756_MOESM4_ESM.docx]

**Article title:** Living with Faecal Incontinence: A qualitative investigation of patients experiences and preferred outcomes through semi-structured interviews

**Journal name:** Quality of Life research

**Author names:** S.L. Assmann, S.O. Breukink , D. Keszthelyi, M.L. Kimman

**Corresponding author:** S.L. Assmann, [s.assmann@maastrichtuniversity.nl](mailto:s.assmann@maastrichtuniversity.nl), Maastricht University, The Netherlands

**Online resource 4: Research team background**

**Interviewer**

Name: Assmann, SL

Gender: Female

Position: PhD candidate surgery and gastroenterology department, University of Maastricht

Credentials during interviews: first half as a medical student, second half as an MD

Experience: All projects within the PhD trajectory are related to patients with faecal incontinence. Trained by a senior qualitative researcher (Kimman, M) on interview techniques and qualitative research techniques.

Participants in this study were aware of the position and credentials of interviewer during interviews. During the interviews, the interviewer and interviewee were the only people present in the room.

**Senior researcher**

Name: Kimman, M

Gender: Female

Position: Senior researcher at the Clinical Epidemiology and Medical Technology Assessment department of the Maastricht University Medical Centre.

Credentials: MSc, PhD

Experience: Work and research interest are in patient-reported outcome measures (PROMs), Core Outcome Set (COS) development, quality of life research and health economic evaluation. She has undergone specific training and has extensive experience in qualitative research.

**Gastroenterologist/researcher**

Name: Keszthelyi, D

Gender: Male

Position: Professor department of Gastroenterology, head of department.

Credentials: Professor, MD, PhD

Experience: Extensive experience in treating patients with Faecal incontinence and research within this field.

**Colorectal surgeon/researcher**

Name: Breukink, S.O.

Gender: Female

Position: Associate professor department of Colorectal surgery.

Credentials: Ass. professor, MD, PhD

Experience: Extensive experience in treating patients with Faecal incontinence and research within this field.
